# Do Financial Constraints, Perceived Food Insecurity, and Pro-Environmental Behavior Explain Intentions to Reduce Meat and Fat Consumption in Older Adults? A Preliminary Study

**DOI:** 10.3390/nu18081259

**Published:** 2026-04-16

**Authors:** Marzena Jeżewska-Zychowicz, Robert Gajda, Rafał Kubacki

**Affiliations:** 1 Department of Food Market and Consumer Research, Institute of Human Nutrition Sciences, Warsaw University of Life Sciences (SGGW-WULS), Nowoursynowska 159C, 02-776 Warsaw, Poland; marzena_jezewska_zychowicz@sggw.edu.pl; 2 Department of Human Nutrition, Faculty of Biotechnology and Food Sciences, Wrocław University of Environmental and Life Science, Norwida 25, 50-375 Wroclaw, Poland; 3 Faculty of Physical Education and Sports, Wroclaw University of Health and Sport Sciences, Paderewskiego 35, 51-612 Wroclaw, Poland; rafal.kubacki@awf.wroc.pl

**Keywords:** older adults, food insecurity, financial support, diet change, meat, fat

## Abstract

Background: The consumption of meat and high-fat foods is constantly discussed, with attention to their health and environmental consequences, as well as the barriers to changing current behaviors. Objective: The study aimed to examine how pro-environmental behavior, perceived food insecurity, and financial constraints correlate with intentions to limit meat and fat consumption among older adults. Methods: A cross-sectional study was conducted in 2025 among 475 individuals aged 60 to 92 years. The questionnaire includes scales that enable the calculation of four scores: Meat Reduction, Low Fat, Perceived Food Insecurity, and Lack of Financial Support. Additionally, questions about involvement in pro-ecological behaviors and sociodemographic characteristics were included. Logistic regression analysis was used to examine associations between perceived food insecurity, lack of financial support, and pro-environmental behaviors (independent variables) and intentions to reduce meat (Model 1) and fat (Model 2) (dependent variables). Results: Intentions to limit meat correlated positively with buying food produced in an environmentally friendly way (adjusted OR = 2.05; 95% CI: 1.56, 2.69), not wasting food (adjusted OR = 1.26; 95% CI: 1.05, 1.51), and buying local food (adjusted OR = 1.48; 95% CI: 1.21, 1.82). Intentions to limit fat correlated positively with buying food produced in an environmentally friendly way (adjusted OR = 1.66; 95% CI: 1.27, 2.18) and not wasting food (adjusted OR = 1.45; 95% CI: 1.20, 1.76). No relationships were found between the lack of financial support and intentions to limit meat (*p* = 0.069) and fat (*p* = 0.600). The perceived food insecurity decreased the likelihood of intentions to restrict fat (adjusted OR = 0.45; 95% CI: 0.24, 0.83), but not meat (*p* = 0.387). Conclusions: To better understand why experienced financial constraints did not influence the intention to reduce consumption of meat and high-fat products, further research is needed that focuses on motivation to change and the ability to change behavior among older people. Nevertheless, the results suggest that enhancing pro-environmental behaviors beyond those directly related to meat and fat consumption may facilitate reductions in meat and fat consumption through pro-environmental behavioral spillover.

## 1. Introduction

The increase in average life expectancy in recent decades has resulted in older people constituting an increasingly large proportion of various populations. However, the increase in lifespan is often associated with older adults experiencing limitations in physical and cognitive functioning, which significantly affect their quality of life and healthcare service use [1]. Healthy physical and mental functioning is primarily associated with diet [2,3,4].

Better physical functioning and reduced age-related problems, such as muscle strength and frailty, in older adults are associated with adequate protein intake [5]. The implementation of the increase in protein intake by 1.0 to 1.2 g/kg body weight/day for older adults aged 65+ years is recommended by several nutrition societies [6,7]. Meat is a good source of high-quality protein [8]. Its consumption is high in the general population and among older people [9], which is of great importance for environmental sustainability [10,11]. Fat is a source of energy and essential fatty acids (EFA) necessary for development, normal function of the nervous and cardiovascular systems, and general health [4]. On the other hand, dietary fat intake is a health risk factor, including obesity and cognitive decline among older populations [12]. Recommendations on fat quality are uniform across various national authorities, which encourage reducing SFA intake and replacing it with MUFAs and PUFAs [13,14].

Familiarity with recommendations regarding meat and fat consumption and willingness to use them may support a positive dietary change in older people; however, other factors, including financial constraints [15,16], perception of food security [16,17,18], beliefs (for example, disbelief of meat impact on the environment) [19], and engagement in some sustainable practices among older people [20,21] may significantly influence meat and fat intake.

Meat consumption and fat consumption are subjects of debate regarding health in old age and sustainability. Meat can help reduce nutritional deficiencies and thus promote human health [2,8]. However, meat has a much greater negative impact on the environment and climate than plant-based foods [22,23]. Hence, consumers are encouraged to limit their intake, especially red meat, for the benefit of both their health and the environment [24]. Similar recommendations also address fat consumption, mainly due to its adverse health effects [12] and, to a lesser extent, environmental challenges [25]. Consumption of meat and fat, which serve as the primary sources of protein and energy in Poles’ habitual diet, becomes more critical under financial constraints [26,27]. For older people with financial constraints, particularly those experiencing energy deficiencies, meeting their basic needs, i.e., food and accommodation, is the most crucial goal before considering environmental sustainability [28]. Moreover, at the individual level, the economic situation of each person can significantly influence their perception of security, behaviors, and decisions in the food sphere, including those referring to environmental sustainability [16,29].

So far, dietary patterns among older individuals, with an emphasis on meat consumption and fat intake, and their determinants have not been sufficiently researched [30]. Although available data indicate low and declining meat consumption among older adults due to poverty [31], there remains considerable uncertainty about meat and fat consumption amid existing financial constraints and food insecurity [15,16]. Environmental motives are already attractive to Western meat eaters (primarily young, eco-minded women) who adopt certain strategies to reduce their meat consumption [32]. However, it is now known that factors other than environmental motives have a greater direct influence on meat consumption practices [33]. Beyond environmental motives, current environmentally friendly behaviors may help limit meat and fat consumption, but knowledge of this relationship is limited.

Taking the above into account, the study aimed to examine how pro-environmental behavior, perceived food insecurity, and lack of financial support correlate with intentions to limit meat and fat consumption among older adults. The following hypotheses were formulated: (1) experiencing both a lack of financial support and food insecurity correlates with intentions to limit meat consumption but not fat consumption; (2) pro-environmental behaviors correlate more strongly with intentions to reduce meat consumption than with those to reduce fat consumption. Understanding the relationships among financial constraints, food insecurity, pro-environmental behaviors, and older adults’ intentions to limit meat and fat consumption may help develop strategies to meet protein and fat recommendations for aging while considering environmental sustainability.

## 2. Materials and Methods

### 2.1. Study Design and Sample

A cross-sectional study was conducted in April and May 2025 among 500 individuals aged 60 to 92 years (mean age, 71.5 ± 6.14 years) from the Lower Silesia region of Poland. The sample was obtained through non-probabilistic convenience sampling, with participants selected based on their accessibility and availability. In collaboration with five senior organizations in Wroclaw, Poland as well as the City and Commune Offices in Siechnice, Poland; Święta Katarzyna, Poland and Strzegom, Poland researchers invited individuals meeting the inclusion criteria to participate in the study. The criteria for inclusion in the group were being 60 years of age or older and residing in a community. The exclusion criteria were as follows: lack of understanding of the questions and lack of independence in completing the questionnaire (cognitive deficits); visual or hearing impairments (sensory deficits); lack of good knowledge of the Polish language; living in the community and simultaneously using day care homes (semi-institutional nature of the residence). The questionnaires with missing data were excluded from the sample. Non-responses resulted from the interview being terminated due to the respondent’s refusal. As a result, the study group consisted of 475 individuals.

A pilot study was conducted among 30 people at a selected senior club in Wroclaw, Poland. Based on feedback from pilot participants, minor adjustments were made to the questionnaire. However, these changes did not apply to the questions used in the presented analyses. One of the researchers was present during the interview (both during the pilot and the main study) and verified that the study group met the exclusion criteria.

Table 1 displays the characteristics of the study sample. Among 475 respondents, 77.3% were women and 22.3% were men. The average age was 71.5 years (SD = 6.13). A greater proportion of respondents (67.6%) lived in urban areas than in rural areas (32.4%). About half of the study group (49.5%) had completed secondary education and reported having enough for their daily needs but needing to save for larger purchases (50.9%).

The study was conducted in accordance with the Declaration of Helsinki. Participation in the survey was voluntary. Informed consent was obtained from all participants. The study was approved by the Rector’s Committee for Research Ethics of the Wrocław University of Environmental and Life Sciences on 14 April 2025, following an opinion on the research project’s compliance with ethical principles (Resolution No. N0N00000.0020.1.5.5.2025).

### 2.2. Questionnaire

The Sustainable Healthy Eating Scale [SHE] [34] was used to assess the intentions to reduce meat and fat consumption. The questionnaire included statements from the Meat Reduction subscale (‘Pulses replace meat in my cooking’, ’I try to eat as many pulses as possible to reduce meat consumption’, ‘I try to eat as much plant–protein source food products as possible, e.g., pulses’, ‘I avoid eating meat’) and Low Fat subscale (‘Whenever possible, I choose low fat food products,’ ‘I choose low fat food products’, ‘I avoid food products containing lots of fat’). Opinions on these statements were presented on a 5-point Likert scale with responses: definitely not (1), rather not (2), neither yes nor no (3), rather yes (4), and definitely yes (5). Cronbach’s alpha = 0.784 for Meat Reduction and Cronbach’s alpha = 0.852 for Low Fat confirmed the scales’ reliability. Then, after summing up the scores for individual statements, a subscale mean was calculated, ranging from 1 to 5. A higher score on the scale indicates a greater willingness to reduce meat/fat consumption.

Three statements from SHE were used to assess the pro-environmental behaviors of respondents: ‘I buy food produced in an environmentally friendly way’, ‘I don’t waste food’, and ‘I buy local food’. Opinions on these statements were presented on a 5-point Likert scale with responses: definitely not (1), rather not (2), neither yes nor no (3), rather yes (4), and definitely yes (5). The three items were analyzed separately because satisfactory reliability was not established for the construct they compose (Cronbach’s alpha = 0.341).

The questionnaire included two questions about perceived food security over the last year due to limited financial resources: (1) In the last year, was there insufficient food in your household because of a lack of money? (2) In the last year, did you have to eat less because of a lack of money? Responses were provided on a 3-point scale: never (1), sometimes (2), and often (3). The two items were analyzed together because the construct composed of them showed satisfactory reliability (Cronbach’s alpha = 0.772). Individuals who answered “never” to both questions were included in the “perceived food security” category, while the remaining individuals were included in the “perceived food insecurity” category.

Two questions were used to assess the respondent’s use of financial support: (1) ‘Do you receive financial aid from your family (including those living in the same household)?’ (2) ‘Do you use social welfare centers due to financial problems?’ Responses were provided on a 4-point scale: there is no need for this, because my financial situation is satisfactory (1); no, even though I have financial problems (2); yes, because I have financial problems (3); yes, even though I do not have financial problems (4). Cronbach’s Alpha coefficient confirmed the scale’s reliability (0.783). Then, the answer “no, even though I have financial problems” was assigned 1 point, while the remaining answers were assigned 0 points. After summing the scores, individuals who scored 1 point or 2 points were classified as not receiving financial support despite experiencing financial problems (‘Lack of Financial Support’). The remaining individuals who did not experience financial problems or receive financial support constituted the second group.

To characterize the study group, questions were used on socio-demographic characteristics (gender, age, education, place of residence), financial situation, and family characteristics (family status)—Table 1.

### 2.3. Statistical Analysis

Descriptive statistics were used to present the demographic characteristics of the sample and the results from the Meat Reduction, Low Fat, Perceived Food Security, and Lack of Financial Support scales. The reliability of the scales was assessed using Cronbach’s alpha, with values of 0.70 or higher considered acceptable [35].

Median was used as a cut-point to identify two categories based on the Meat Reduction and Low Fat scores: those with a low score (below the median), indicating minor restrictions on meat/fat consumption, and those with a high score (at or above the median), indicating more significant limits on meat/fat consumption. When presenting the results, except for the logistic regression results, the variables describing pro-ecological behaviors were also dichotomized, with the answers definitely not (1), rather not (2), neither yes nor no (3) marked as “No” (no behavior), and the answers rather yes (4), and definitely yes (5) as “Yes” (behavior occurs).

The normality of continuous variable distributions was assessed using the Kolmogorov–Smirnov test. The chi-square test of independence was used to determine differences between these groups. A significance level of *p* < 0.05 was considered significant.

Logistic regression analysis was used to verify associations between perceived food insecurity (ref. perceived food security), lack of financial support (ref. no need or using support), pro-environmental behaviors (I buy food produced in an environmentally friendly way; I don’t waste food, and I buy local food (independent variables), and Meat Reduction (Model 1) and Low Fat (Model 2) as dichotomous variables (dependent variables). Odds ratios (OR) represented the probability of belonging to a group of individuals declaring intentions to limit meat consumption (Model 1) and to a group with intentions to limit fat consumption (Model 2). The reference groups (OR = 1.00) did not report intentions to limit meat or fat consumption. Both models were developed in two versions: one crude and one adjusted for gender, place of residence, education, family status, age, and self-reported economic situation. The selection of covariates was made a priori, using available empirical evidence on important confounding factors [35]. Wald’s test was used to assess the significance of ORs.

All analyses were performed using IBM SPSS Statistics, version 29.0 (IBM Corp, Armonk, NY, USA).

## 3. Results

### 3.1. Perceived Food Insecurity, Lack of Financial Support, and Selected Pro-Environmental Behaviors in the Study Sample

In the study group, 16.8% of participants reported a need for financial support (Table 2 and Table A1, Appendix A). Lack of financial support was reported by more individuals with less than a secondary education (36.0%), those living alone (20.8%), and individuals who described their situation as “I live very modestly” (63.5%).

Food insecurity was reported by 20% of the study sample. It was reported by more individuals aged 60–65 (32.4%), and 71–75 (24.3%), those with education below secondary level, those living in smaller towns and in rural areas reported food insecurity (25.5% and 22.1%, respectively), and individuals in the worst financial situation (63.9%) (Table 2).

In the study group, 76.0% reported purchasing environmentally friendly food, and 85.3% stated that they did not waste food. More than half of the respondents (55.8%) declared purchasing local food (Table 2). Purchase of environmentally friendly food was reported by more women (80.2%) and people with secondary education (82.1%). A higher proportion of men (91.5%) and individuals with higher levels of education (91.9%) reported not wasting food. A higher percentage of individuals in small towns (65.0%) and those living alone (61.9%) reported purchasing local food (Table 2).

### 3.2. Predictors of Intentions to Limit Consumption of Meat and Fat

Descriptive statistics for the Low Fat and Meat Reduction scores are presented in Table A2 and Table A3 (Appendix A). In the study group, a higher median score was observed for limiting fat intake (4.0) than for limiting meat intake (2.7).

A higher Low Fat score (above the median signifying greater willingness to reduce fat consumption) was achieved by more women than men and by more individuals who perceived food security than those who perceived food insecurity. Moreover, a higher Low-fat score was achieved by more individuals who reported buying environmentally friendly food, not wasting food, and buying local food than by those who did not report such behaviors. A higher Meat Reduction score (above the median signifying greater willingness to reduce meat consumption) was achieved by more individuals who reported buying environmentally friendly food, not wasting food, and buying local food than by those who did not report such behaviors (Table 3).

### 3.3. Predictors of Intentions to Limit Meat and Fat Consumption

Perceived food insecurity reduced the likelihood of declaring intentions to limit fat consumption by 55% (Model 1). In contrast, perceived food insecurity had no predictive effect on limiting meat consumption (Model 2) (Table 4). A 1-point higher score, indicating purchasing environmentally friendly food, increased the likelihood of declaring intentions to reduce fat consumption by 66% and meat consumption by 105%. Declaring no food waste increased the likelihood of intentions to limit fat consumption by 45% and intentions to limit meat consumption by 26%. The predictive effect of purchasing local food was only observed for intentions to reduce meat consumption. The likelihood of declaring intentions to limit meat increased by 48% when local food purchases increased by 1 point. Lack of financial support was not a predictor of intentions to reduce fat and meat consumption.

## 4. Discussion

This study aimed to investigate the relationship between pro-environmental behavior, financial constraints, perceived food insecurity, and intentions to limit meat and fat intake among older adults. The results show that declared intentions to limit meat and fat consumption correlated positively with pro-environmental behaviors such as buying food produced in an environmentally friendly way and not wasting food, and, in the case of meat, also with buying local food. No relationship was found between experiencing a lack of financial support when experiencing financial problems and intentions to limit meat and fat consumption. Perceived food insecurity decreased the likelihood of reporting intentions to restrict fat intake, but there was no association with intentions to limit meat intake.

Our study did not confirm the first hypothesis that both lack of financial support and food insecurity correlate with intentions to limit meat consumption, but not fat consumption. Previous studies have shown that meat prices and meat availability (e.g., distance to shopping centers) are the main barriers to meat consumption [36,37]. Moreover, wealth was positively correlated with meat consumption [38]. The lack of association between experiencing a lack of financial support and changes in the consumption of meat in the group of older adults may indicate the importance of other factors, including attitudes, values, subjective norms, culinary skills, but also habits and traditions [39,40], which were not taken into account in the study. Tradition, social norms, and lack of culinary skills make it particularly difficult to change behaviors related to meat consumption [32]. Moreover, the reluctance to change may be linked to the high status of meat in many cultures and its perception as essential to social life [32,41], rather than solely to its nutritional and taste values [42].

Moreover, experiencing financial difficulties is widely considered to make it difficult, if not impossible, to change eating habits [43,44]. Research findings to date indicate that low-income groups typically face greater barriers to consuming plant-based foods [45,46] and are more likely to choose cheaper meat products than those in higher-income groups [45]. The main barriers low-income individuals face in accessing food, in addition to perceived costs, culinary knowledge, and taste, are cultural patterns that favor meat consumption over plant-based foods [47]. The high price of meat may encourage people to limit their consumption when their financial situation deteriorates, and there is no external support [48]. Still, it may also encourage the purchase of cheaper meat products [45].

The lack of intention to reduce meat and fat consumption can also be seen as an attempt to protect current levels of consumption as financial conditions deteriorate. Such behavior has been previously reported in Poland [49]. Fat consumption, as a relatively cheap source of energy in the diet [25], may be easier to maintain, especially since fat is a feature of traditional Polish cuisine. Therefore, it can be assumed that the long-established trait of the Polish diet, namely high fat content, as well as the relatively lower price of such foods in situations of food insecurity, increased the usefulness of high-fat foods in meeting needs. This is confirmed by the almost two-fold lower odds of intending to reduce fat consumption in the presence of perceived food insecurity. Similarly, as in other studies, perceived food insecurity is a barrier to dietary change [50,51].

The study’s results have not confirmed a link between perceived insecurity and intentions to reduce meat consumption. Perceiving meat as a safe product can encourage continued consumption. However, the measurement itself may also be important. The reduction in meat consumption in SHE is primarily attributed to increased consumption of pulses and other plant-based foods [34]. This type of food remains relatively unpopular in many populations, including among older individuals [52,53,54]. A study in the Netherlands found that older adults were more likely to prefer a smaller meat portion than to replace meat with something else [54,55]. The elderly population may therefore be in conflict over meat versus pulses, a problematic issue to resolve for many reasons. Although both product groups are typical of Polish cuisine, meat has always been considered a high-status food. Limiting its consumption, especially among older adults, may be seen as a sign of lower social standing. Furthermore, the skill and convenience of preparing food may be a factor when comparing meat to pulses [56,57].

Greater engagement in pro-environmental behaviors was positively correlated with both intentions to reduce meat and fat consumption. However, a stronger predictive effect was observed for intentions to reduce meat consumption, confirming partly hypothesis 2. A higher Meat Reduction score was observed for individuals who reported purchasing environmentally friendly food and those who purchased locally sourced food. The most substantial predictive effect was observed for buying food produced in an environmentally friendly way, and a significant effect was also observed for the other two behaviors. These results are consistent with other studies indicating pro-environmental behavioral spillover [58,59]. Purchasing food produced in an environmentally friendly way and not wasting food increased the likelihood of reducing fat consumption, but to a lesser extent than reducing meat consumption. Purchasing locally produced food did not correlate with such intentions. Thus, the second hypothesis was verified.

### 4.1. Strengths and Limitations

A strength of the study is its approach to explaining planned changes in the consumption of meat and fat-rich foods, which are important sources of protein and energy in the diet of older adults. Taking into account financial status (experiencing financial constraints and perceived food insecurity) on the one hand, and the behavioral construct (selected pro-environmental behaviors) on the other, provides new insights into the complex determinants of engagement with healthy, sustainability-oriented food choices in older adults. The application of the results in the development of educational and intervention strategies should also be noted.

Several limitations of this study should be acknowledged. All data obtained from older adults were self-reported, raising concerns about accuracy and potential social desirability effects. While the findings suggest that financial problems experienced by older adults without external support do not influence intentions to limit meat and fat consumption, a different relationship is also possible. Older adults may not want to change and have not declared any intention to change their meat and fat intake, but changes can occur depending on current circumstances [27]. Second, the study employed a cross-sectional design, capturing data at a single point in time, which enables cause-and-effect inference. Moreover, the study was conducted in a single cultural context—Poland, a country with a strongly meat-centric food culture [26,60]. While this focus yields valuable insights into Polish older adults, the generalizability of the findings to other cultures may be limited.

Although convenience sampling used in the study offers several advantages (cost-effective, time-saving), it also has significant drawbacks [61,62]. It may not accurately reflect the characteristics of the entire population. Our study group lacked gender balance. Therefore, conclusions may be biased, reflecting the specific attributes of the sample rather than the broader population of interest. Such sampling can lead to overrepresentation of certain groups or opinions, thus biasing the results. Furthermore, researchers may unwittingly select participants who appear most willing or eager to participate, further exacerbating the bias. Such biases can significantly affect the reliability and validity of study results, limiting their representativeness and applicability to broader contexts.

Convenience sampling is also associated with non-response bias [62]. The inability to compare the characteristics of study participants with those of the remaining population aged 60+ prevents the estimation of non-response bias. The recruitment conducted by researchers limited cases of refusal to participate in the study. However, rejected questionnaires (25) due to interview interruption led to non-response bias. The lack of information on the socio-demographic characteristics of these individuals prevented an assessment of the likelihood of non-response bias by comparing the characteristics of respondents and non-respondents. The presence of an interviewer during the interview may, however, have influenced responses (e.g., social desirability bias), especially regarding the experience of financial constraints and expectations of external support.

The dichotomization of continuous variables is also a limitation of the study. The loss of information reduces the statistical power to detect an association between variables [63]. It can also increase the risk of a positive result being a false positive. Dichotomizing continuous variables can seriously underestimate the range of variability in group scores. Individuals close to the cutoff point but on opposite sides are characterized as very different rather than very similar [64,65,66].

Another limitation of the study was the use of non-validated scales to assess pro-environmental behaviors, social support, and financial support. Confirming their reliability does not eliminate measurement error, especially given the small number of statements used to identify individual constructs. Furthermore, many confounders and mediators (e.g., cooking skills, habits, and health literacy) are unmeasured, which may influence intentions to reduce meat and fat consumption.

### 4.2. Directions for Future Research

Building on the results and the limitations, several avenues for future research emerge. Understanding why financial constraints experienced by people who did not receive financial support did not determine their intention to change their consumption of meat and high-fat foods requires further research that will additionally assess the motivation to change, the ability to make a change, and the factors facilitating the change [67]. Furthermore, future studies should consider different types of meat, including red and white meat, and should also account for processing levels. Previous studies have shown that older individuals tend to reduce their consumption of red meat [68] or eat less red meat [69]. Since in the study group, pro-ecological behaviors related to intentions to reduce meat and fat consumption showed differences after taking into account socio-demographic characteristics, further research should focus on these differences, which may facilitate the preparation of strategies addressed to specific groups of older people, e.g., distinguished based on place of residence, but also on financial situation. Addressing these areas would contribute to a more comprehensive understanding of how to promote dietary transitions that achieve nutritional and environmental goals, while enhancing the quality of life of older adults.

## 5. Conclusions

The results have shown that declared intentions to limit meat and fat consumption correlated positively with pro-environmental behaviors such as buying food produced in an environmentally friendly way and not wasting food, and, in the case of meat, also with buying local food. No relationship was found between experiencing a lack of financial support and intentions to limit meat and fat consumption. Perceived food insecurity decreased the likelihood of reporting intentions to restrict fat intake, but there was no association between perceived food insecurity and intentions to limit meat intake.

Considering today’s health challenges in older populations, there is an urgent need to adapt dietary behaviors to nutritional recommendations, especially among those in meat-centric food cultures. Enhancing pro-environmental behaviors beyond those directly related to meat and fat consumption may facilitate reductions in meat and fat consumption through pro-environmental behavioral spillover, as our study confirms. However, in this population group, continuous monitoring of consumption levels, particularly of meat, is necessary to ensure adequate intake of protein and other essential dietary components.

Despite the limitations of the methodological approach, including a non-representative study group, the results contribute to a better understanding of the complex interplay among older adults’ intentions to limit meat and fat consumption, their experiences of financial problems and food insecurity, and their engagement in pro-environmental behaviors. This integration provides a more nuanced understanding of how these characteristics affect their engagement with healthy, sustainability-oriented food choices.

## Figures and Tables

**Table 1:** Sociodemographic characteristics of the study sample.

Characteristics		% (*N*) *
Total		100.0 (475)
Gender	Woman	77.7 (369)
	Man	22.3 (106)
Age	60–65 years	15.6 (74)
	66–70 years	34.1 (162)
	71–75 years	28.6 (136)
	Above 75 years	21.7 (103)
Education	Primary	3.4 (16)
	Vocational	18.5 (88)
	Secondary	49.5 (235)
	Higher	28.6 (136)
Place of residence	Rural area	32.4 (154)
	A town with fewer than 100,000 inhabitants	28.8 (137)
	City with more than 100,000 inhabitants	38.8 (184)
Family status	I live alone	42.5 (202)
	I live with my husband/wife	45.7 (217)
	I live with my family, without my partner	4.9 (23)
	I live with my husband/wife and my family	6.9 (33)
Self-reported financial situation	I live in poverty	0.0 (0)
	I live very modestly	12.9 (61)
	I live moderately	50.9 (242)
	I live well	31.8 (151)
	I live very well	4.4 (21)

* *N*—number of respondents.

**Table 2:** Perceived food insecurity, lack of financial support, and pro-environmental behavior in the study group, taking into account socio-demographic characteristics.

	Lack of Financial Support % (*n* *)	Perceived Food Insecurity % (*n*)	Pro-Environmental Behavior		
			I Buy Food Produced in an Environmentally Friendly Way % (*n*)	I Don’t Waste Food % (*n*)	I Buy Local Food % (*n*)
Total % (*n*) *	16.8 (80)	20.0 (95)	76.0 (361)	85.3 (405)	55.8 (265)
**Gender**	*p* = 0.153 **	*p* = 0.741	*p* < 0.001	*p* = 0.040	*p* = 0.071
Women (*n* = 369)	18.2 (67)	20.3 (75)	80.2 (296)	83.5 (308)	58.0 (214)
Men (*n* = 106)	12.3 (13)	18.9 (20)	61.3 (65)	91.5 (97)	48.1 (51)
**Age**	*p* = 0.744	*p* = 0.003	*p* = 0.282	*p* = 0.918	*p* = 0.350
60–65 years (*n* = 74)	18.9 (14)	32.4 (24)	82.4 (61)	87.8 (65)	64.9 (48)
66–70 years (*n* = 162)	14.8 (24)	13.6 (22)	76.5 (124)	84.6 (137)	52.5 (85)
71–75 years (*n* = 136)	16.2 (22)	24.3 (33)	76.5 (104)	85.3 (116)	55.9 (76)
Above 75 years (*n* = 103)	19.4 (20)	15.5 (16)	69.9 (72)	84.5 (87)	54.4 (56)
**Education**	*p* = 0.006	*p* < 0.001	*p* = 0.002	*p* = 0.022	*p* = 0.100
Lower than secondary (*n* = 104)	26.0 (27)	36.5 (38)	75.0 (78)	79.8 (83)	55.5 (58)
Secondary (*n* = 235)	16.6 (39)	17.4 (41)	82.1 (193)	83.8 (97)	60.0 (141)
Higher (*n* = 136)	10.3 (14)	11.8 (16)	66.2 (90)	91.9 (125)	48.5 (66)
**Place of residence**	*p* = 0.118	*p* = 0.030	*p* = 0.119	*p* = 0.693	*p* = 0.033
Rural area (*n* = 154)	21.4 (33)	22.1 (34)	81.8 (126)	83.8 (129)	50.6 (78)
A town with fewer than 100,000 inhabitants (*n* = 137)	12.4 (17)	25.5 (35)	73.7 (101)	84.7 (116)	65.0 (89)
City with more than 100,000 inhabitants (*n* = 184)	16.3 (30)	14.1 (26)	72.8 (134)	87.0 (160)	53.3 (98)
**Family status**	*p* = 0.048	*p* < 0.001	*p* = 0.741	*p* = 0.103	*p* = 0.021
I live alone (*n* = 202)	20.8 (42)	28.7 (58)	75.2 (152)	82.2 (166)	61.9 (125)
I live with others (*n* = 273)	13.9 (38)	13.6 (37)	76.6 (209)	87.5 (239)	51.3 (140)
**Self-reported financial situation**	*p* < 0.001	*p* < 0.001	*p* = 0.839	*p* = 0.323	*p* = 0.105
I live very modestly (*n* = 61)	63.9 (39)	63.9 (39)	73.8 (45)	85.2 (52)	52.5 (32)
I live moderately (*n* = 242)	14.9 (36)	20.7 (50)	75.6 (183)	83.1 (201)	52.1 (126)
I live well or very well (*n* = 172)	2.9 (5)	3.5 (6)	77.3 (133)	88.4 (152)	62.2 (107)

* number of participants; ** significance level, Chi-square test.

**Table 3:** Intention to limit fat and meat consumption in the study group, taking into account selected sociodemographic characteristics and pro-environmental behaviors (%).

	Low Fat		Meat Reduction	
	≤Median (*n* * = 277)	>Median (*n* = 198)	≤Median (*n* = 240)	>Median (*n* = 235)
**Gender**	*p* = 0.002 **		*p* = 0.902	
Women (*n* = 369)	54.5 (201)	45.5 (168)	50.7 (187)	49.3 (182)
Men (*n* = 106)	71.7 (76)	28.3 (30)	50.0 (53)	50.0 (53)
**Perceived food insecurity**	*p* = 0.007		*p* = 0.819	
No (*n* = 380)	55.3 (210)	44.7 (170)	50.8 (193)	49.2 (187)
Yes (*n* = 95)	70.5 (67)	29.5 (28)	49.5 (47)	50.5 (48)
**Buying food produced in an environmentally friendly way**	*p* < 0.001		*p* < 0.001	
No (*n* = 114)	74.6 (85)	25.4 (29)	76.3 (87)	23.7 (27)
Yes (*n* = 361)	53.2 (192)	46.8 (169)	42.4 (153)	57.6 (208)
**No food waste**	*p* = 0.016		*p* = 0.006	
Yes (*n* = 405)	56.0 (227)	44.0 (178)	47.9 (194)	52.1 (211)
No (*n* = 70)	71.4 (50)	28.6 (20)	65.7 (46)	34.3 (24)
**Buying local food**	*p* = 0.002		<0.001	
No (*n* = 210)	66.2 (139)	33.8 (71)	64.8 (136)	35.6 (74)
Yes (*n* = 265)	52.1 (138)	47.9 (127)	39.2 (104)	60.8 (161)

* Number of participants; ** significance level, Chi-square test.

**Table 4:** Determinants of intentions to limit consumption of meat and fat in the study sample—results of logistic regression models.

	Model	B *	Exp(B) *	95% Confidence Interval for Exp(B) *		*p*-Value **
				Lower	Upper	
**Model 1—Low Fat**						
Perceived food insecurity (ref. perceived food security)	Crude	−0.70	0.50	0.28	0.87	0.015
	Adjusted ***	−0.80	0.45	0.24	0.83	0.011
Lack of financial support (ref. no need or using support)	Crude	0.72	1.08	0.59	1.95	0.811
	Adjusted	0.18	1.20	0.61	2.36	0.600
I buy food produced in an environmentally friendly way	Crude	0.55	1.74	1.34	2.26	<0.001
	Adjusted	0.51	1.66	1.27	2.18	<0.001
I don’t waste food	Crude	0.34	1.41	1.17	1.71	<0.001
	Adjusted	0.37	1.45	1.20	1.76	<0.001
I buy local food	Crude	0.15	1.17	0.96	1.42	0.132
	Adjusted	0.13	1.14	0.93	1.39	0.215
**Model 2—Meat Reduction**						
Perceived food insecurity (ref. perceived food security)	Crude	0.34	1.40	0.80	2.438	0.239
	Adjusted	0.26	1.30	0.72	2.365	0.387
Lack of financial support (ref. no need or using support)	Crude	−0.46	0.63	0.35	1.15	0.131
	Adjusted	−0.59	0.55	0.29	1.05	0.069
I buy food produced in an environmentally friendly way	Crude	0.69	2.00	1.54	2.60	<0.001
	Adjusted	0.72	2.05	1.56	2.69	<0.001
I don’t waste food	Crude	0.24	1.28	1.07	1.52	0.007
	Adjusted	0.23	1.26	1.05	1.51	0.013
I buy local food	Crude	0.36	1.43	1.18	1.74	<0.001
	Adjusted	0.40	1.48	1.21	1.82	<0.001

* OR—point estimate (B, Exp(B), 95% confidence intervals; ** *p*-value—significance level of the Wald’s test; *** adjusted for gender, place of residence, education, living status, age, and self-reported economic situation.

## Data Availability

Data presented in this study are available on request from the corresponding author. The data are not publicly available due to the ongoing nature of the study.

## Appendix A

**Table A1:** Percentage distribution of responses for variables included in the analysis.

Variables	Responses				
**Meat Reduction scale**	Definitely not (1)	Rather not (2)	Neither yes nor no (3)	Rather yes (4)	Definitely yes (5)
Pulses replace meat in my cooking	9.9 (47)	48.2 (229)	9.3 (44)	25.3 (120)	7.3 (35)
I try to eat as many pulses as possible to reduce meat consumption	5.5 (26)	37.6 (179)	15.2 (72)	27.6 (131)	14.1 (67)
I try to eat as many plant-protein source food products as possible, e.g., pulses	2.5 (12)	19.4 (92)	13.3 (63)	49.0 (233)	15.8 (75)
I avoid eating meat	15.2 (72)	56.6 (269)	5.1 (24)	18.7 (89)	4.4 (21)
**Low-fat scale**	Definitely not (1)	Rather not (2)	Neither yes nor no (3)	Rather yes (4)	Definitely yes (5)
Whenever possible, I choose low-fat food products	3.2 (15)	14.5 (69)	6.5 (31)	48.0 (228)	27.8 (132)
I choose low-fat food products	2.1 (10)	15.8 (75)	5.9 (28)	45.7 (217)	30.5 (145)
I avoid food products containing lots of fat	2.1 (10)	14.5 (69)	6.1 (29)	44.0 (209)	33.3 (158)
**Pro-environmental behaviors**	Definitely not (1)	Rather not (2)	Neither yes nor no (3)	Rather yes (4)	Definitely yes (5)
I buy food produced in an environmentally friendly way	0.6 (3)	5.9 (28)	17.5 (83)	47.8 (227)	28.2 (134)
I don’t waste food	5.1 (24)	8.0 (38)	1.7 (8)	29.1 (138)	56.1 (267)
I buy local food	3.8 (18)	21.9 (104)	18.5 (88)	43.2 (205)	12.6 (60)
**Financial support**	There is no need for this, because my financial situation is satisfactory (1)	No, even though I have financial problems (2)	Yes, because I have financial problems (3)	Yes, even though I do not have financial problems (4)	X
Do you receive financial aid from your family (including those living in the same household?	75.7 (359)	10.7 (51)	4.8 (23)	8.8 (42)	
Do you use social welfare centers due to financial problems?	81.1 (386)	6.1 (28)	12.8 (61)	0.0 (0)	
**Perceived Food Security**	Never	Sometimes	Often	X	X
In the last year, was there insufficient food in your household because of a lack of money?	83.6 (397)	15.2 (72)	1.3 (6)		
In the last year, did you have to eat less because of a lack of money?	85.1 (404)	13.9 (66)	1.0 (5)		

**Table A2:** Descriptive statistics for the Low Fat score and its determinants.

Low Fat Score		Total	Gender *		Perceived Food Security *		I Buy Food Produced in an Environmentally Friendly Way *		I Don’t Waste Food **		I Buy Local Food *	
			Women	Men	Yes	No	No	Yes	No	Yes	No	Yes
Total % (*N*)		100.0 (475)	77.7 (369)	22.3 (106)	80.0 (380)	20.0 (95)	24.0 (114)	76.0 (361)	14.7 (70)	85.3 (405)	44.2 (210)	55.8 (265)
Mean		3.9	3.9	3.6	4.0	3.6	3.5	4.0	3.6	3.9	3.7	4.0
95% confidence interval for the mean	Lower limit	3.8	3.8	3.4	3.9	3.3	3.3	3.9	3.3	3.8	3.6	3.9
	Upper limit	4.0	4.0	3.8	4.0	3.8	3.6	4.1	3.8	4.0	3.8	4.1
Median		4.0	4.0	4.0	4.0	4.00	3.7	4.0	4.0	4.0	4.0	4.0
Variance		0.90	0.90	0.83	0.81	1.18	0.97	0.81	0.96	0.88	0.95	0.83
Std. dev.		0.95	0.95	0.91	0.90	1.09	0.98	0.90	0.98	0.94	0.98	0.91
Minimum		1.0	1.0	1.3	1.0	1.3	1.3	1.0	1.3	1.0	1.3	1.0
Maximum		5.0	5.0	5.0	5.0	5.0	5.0	5.0	5.0	5.0	5.0	5.0
Range		4.0	4.0	3.7	4.0	3.7	3.7	4.0	3.7	4.0	3.6	4.0
Interquartile range		1.3	1.3	1.3	1.3	1.3	1.6	1.0	1.4	1.3	1.3	1.0
Skewness		−0.873	−1.007	−0.533	−0.923	−0.567	−0.373	−1.087	−0.750	−0.907	0.527	−1.208
Kurtosis		0.046	0.351	−0.480	0.174	−0.544	−0.792	0.717	−0.345	0.146	−0.610	1.047

* *p* < 0.001, ** *p* = 0.003 (U Mann–Whitney test).

**Table A3:** Descriptive statistics for the Meat Reduction score and its determinants.

Meat Reduction Score		Total	Gender		Perceived Food Security		I Buy Food Produced in an Environmentally Friendly Way *		I Don’t Waste Food		I Buy Local Food *	
			Women	Men	Yes	No	No	Yes	No	Yes	No	Yes
Total % (*N*)		100.0 (475)	77.7 (369)	22.3 (106)	80.0 (380)	20.0 (95)	24.0 (114)	76.0 (361)	14.7 (70)	85.3 (405)	44.2 (210)	55.8 (265)
Mean		2.9	2.9	2.9	2.9	3.1	2.4	3.1	2.8	3.0	2.7	3.1
95% confidence interval for the mean	Lower limit	2.9	2.9	2.7	2.8	2.9	2.3	3.0	2.6	2.9	2.6	3.0
	Upper limit	3.0	3.1	3.0	3.0	3.2	2.6	3.2	3.0	3.1	2.8	3.3
Median		2.7	2.7	2.9	2.8	3.0	2.2	3.0	2.5	3.0	2.5	3.0
Variance		0.77	0.79	0.69	0.78	0.72	0.59	0.72	0.71	0.78	0.59	0.81
Std. dev.		0.88	0.89	0.83	0.88	0.85	0.77	0.85	0.84	0.88	0.77	0.90
Minimum		1.3	1.3	1.3	1.3	1.5	1.3	1.3	1.3	1.3	1.3	1.3
Maximum		5.0	5.0	5.0	5.0	5.0	5.0	5.0	5.0	5.0	5.0	5.0
Range		3.75	3.75	3.75	3.75	3.50	3.75	3.75	3.75	3.75	3.75	3.75
Interquartile range		1.25	1.25	1.25	1.25	1.50	0.75	1.25	1.00	1.25	0.75	1.50
Skewness		0.312	0.376	−0.004	0.292	0.476	0.657	0.256	0.701	0.251	0.484	0.095
Kurtosis		−0.494	−0.533	−0.536	−0.471	−0.731	0.669	−0.631	0.132	−0.537	0.229	−0.749

* *p* < 0.001 (U Mann–Whitney test).
